# High-Grade Temporal Ganglioglioma in an Older Adult Woman

**DOI:** 10.7759/cureus.45862

**Published:** 2023-09-24

**Authors:** Isauro Lozano Guzmán, Bayron A Sandoval-Bonilla, Jesús E Falcon Molina, Ricardo Garcia Iturbide, Luis A Castillejo Adalid, Yelitza A Valverde García, Luis A Amaya Morante

**Affiliations:** 1 Neurological Surgery, Hospital de Especialidades del Centro Médico Nacional Siglo XXI, Mexico City, MEX; 2 Neurological Surgery, Hospital de Especialidades del Centro Médico Nacional Siglo XXI, Mexico city, MEX; 3 Pathology, Hospital de Especialidades del Centro Médico Nacional Siglo XXI, Mexico City, MEX

**Keywords:** aphasia, focal-onset seizures, glio-neural tumor, anaplastic ganglioglioma, ganglioglioma

## Abstract

Ganglioglioma (GG) is a WHO-grade 1 glioneuronal neoplasm. It is well differentiated with a slow-growing pattern and is composed of a combination of neoplastic ganglion and glial cells. Anaplastic ganglioglioma (AGG) is an extremely rare malignant variant of ganglioglioma, which is not included in the new WHO classification; however, the term is used to talk about gangliogliomas with data of malignancy. AGGs usually occur in children and young adults and are associated with high recurrence and mortality.

The authors describe the case of a 62-year-old woman with AGG. She presented with cacosmia, vertigo, nausea, and focal-onset seizures with secondary generalization. Magnetic resonance imaging (MRI) revealed an intra-axial lesion in the left temporal lobe. She underwent microsurgical resection guided by electrocorticography (ECoG), and a diagnosis of AGG based on microscopic morphology and immunohistochemical analysis was obtained. She was discharged a few days after surgery with subtotal resection of the lesion, no additional neurological deficit, and adequate seizure control.

AGG is a very rare and poorly studied entity. It is currently a controversial term used to refer to gangliogliomas with signs of malignancy. It occurs mainly in children and young adults with temporal lobe epilepsy. Total resection is the best prognostic factor, given the unknown efficacy of radiotherapy and chemotherapy. In our case, the patient was an adult woman with a subtotal resection followed by concomitant radiotherapy and chemotherapy, obtaining a mean survival similar to that reported in the literature, so it can be thought that there is a benefit obtained with chemotherapy and radiotherapy despite having performed a subtotal resection of the lesion. Further studies are needed to establish clear diagnostic criteria for AGG, and a multicenter database of AGGs is necessary for a better understanding of the pathology and to offer the best treatment and prognosis.

## Introduction

Gangliogliomas (GGs) are very rare mixed glio-neural tumors that represent approximately 1.3% of all primary brain tumors and 0.4% of central nervous system neoplasms [1]. They were first described in 1926 and are classified as World Health Organization (WHO) grade 1 tumors. They arise from glioneuronal precursor cells that are capable of differentiation into glial and neuronal components and manifest typically with epilepsy [2].

Anaplastic ganglioglioma (AGG) is a rare malignant variant that is poorly characterized and, right now, is not included in the new WHO classification but is used to talk about gangliogliomas with data of malignancy [3]. It represents about 1-5% of all gangliogliomas, with an incidence of 0.06 cases per million per year, and is associated with worse local control rates and shorter overall survival [2]. AGG can arise spontaneously or secondary to the malignant transformation of a previous WHO-grade 1 ganglioglioma. The rates of malignant transformation vary from 0.6% to 14.5% [4]. Given that patients are generally children and young adults with a slight preponderance of males, this report represents a rare presentation because of the age group and sex of the patient [5]. The average survival rate is about 24.7 months, making this entity one of the most aggressive in neurological oncology [2,6].

## Case presentation

**Figure 1:**
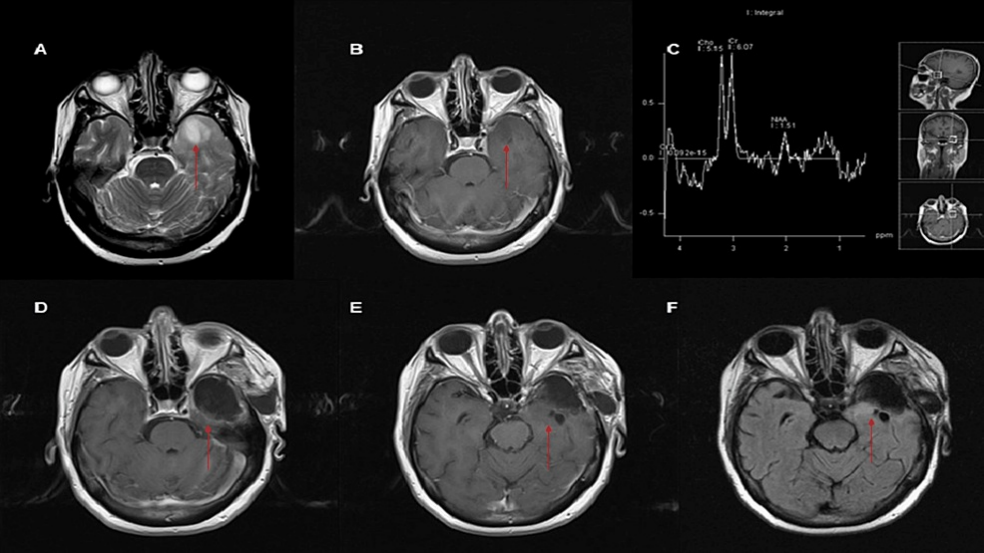
Preoparative and postoperative MRI. (A) Axial MR slice on T2 sequence who shows left temporal lession (arrow). (B) Axial MR slice T1 sequence shows isointense lession on left temporal lobe (arrow). (C) MRI spectroscopy with a peak of choline, N-acetyl aspartate and choline. (D-F) Postoperative MRI on T1 and Flair sequences showing subtotal resection of the lession (arrows). MRI: magnetic resonance imaging.

A 62-year-old woman with a previous history of systemic arterial hypertension and hyperthyroidism treated with hemithyroidectomy and thiamazole was admitted to our hospital. The patient complained of cacosmia, vertigo, nausea, and focal-onset seizures with secondary generalization that began about one month before admission. Previously, a neurologist prescribed levetiracetam 3 g per day and lamotrigine 50 mg twice daily with inadequate seizure control. Neurological examination exhibited global aphasia with memory preservation and no additional neurological deficit. Serum laboratory analyses were normal. A cranial MRI scan revealed an intra-axial and diffuse lesion in the left temporal lobe measuring 15 mm × 20 mm × 19 mm, associated with perilesional edema to rule out infiltration of the neoplasm. The lesion was isointense in T1-weighted and hyperintense in T2-weighted sequences without contrast enhancement. Spectroscopy showed an elevation of creatine and choline with a decrease of N-acetyl aspartate (Figure 1).

Surgical procedure and histopathological analysis

Awake surgery could not be performed because the patient failed to pass preoperative neuropsychology testing to undergo the procedure. We decided to perform the surgery under general anesthesia in a supine position with the head rotated to the right in three-point fixation in the Mayfield clamp. A Yasargil incision and a left pterional craniotomy bone flap were performed. Dura was opened in a C-shaped fashion, and, with the use of neuronavigation and electrocorticography (ECoG), a guided resection was performed to achieve a temporal lesionectomy without changes in the ECoG. Mesial structures were not included to avoid memory impairment, and hemostasis was secured, closing the dura in a watertight fashion.

Intraoperative pathological analysis reported only a low-grade astrocytoma without another specification.

**Figure 2:**
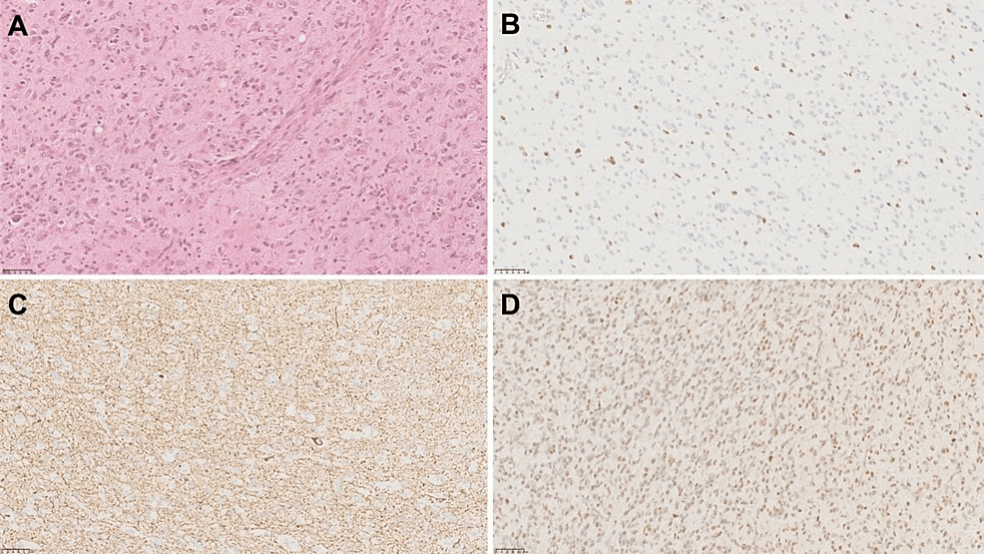
Histopathological analyses was compatible with anaplastic ganglioglioma (×10). (A) H&E, (B) Ki 67 positive, (C) NFP positive, (D) P53 positive. H&E: hematoxylin and eosin; NFP: neurofilament protein.

**Figure 3:**
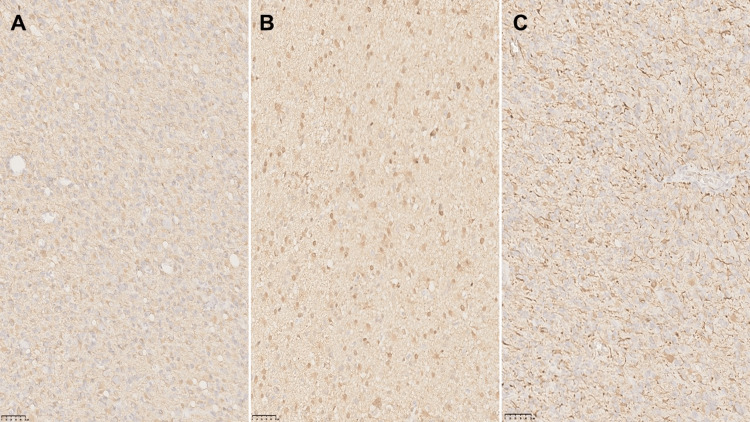
Continuation of histopathological analysis (×10). (A) GFAP positive, (B) S100 positive, (C) WT1 positive. GFAP: glial fibrillary acidic protein; WT1: Wilms’ tumor 1 protein.

The definitive study was described as a lesion of 3.8 cm × 2.6 cm × 1.8 cm, weighing 12 g, light brown on the external surface, lobed, opaque, of soft consistency, with thin blood vessels; on the inner surface, it is light brown, rough, and soft. Histopathology showed an admixture of neoplastic ganglion and glial cells with microvascular proliferation and mitosis. Ganglion cells showed clustering and cytomegaly; some of them showed binucleated forms; and glial cells showed astrocytic morphology. The immunohistochemical analysis showed positivity for glial fibrillary acidic protein (GFAP), Wilms’ tumor 1 protein (WT1), p53, S100 protein, Ki67 (15%), neurofilament protein (NFP), and synaptophysin (Figures 2-3). Neurofilament protein and synaptophysin highlight the neuronal component in gangliogliomas; GFAP and S100 protein highlight the neoplastic glial cell component. Ki-67 is usually limited to the glial component and is typically low in GGs (<5%); in our case, we had a high Ki-67 proliferation index (15%) corresponding to a high-grade lesion. There was no resource to perform CD34, which is usually positive in GGs.

Due to the lack of resources, further molecular analysis could not be possible.

Postoperative course

The patient recovered with no neurological deficit and was discharged six days after surgery. Postoperative MRI showed subtotal resection of the tumor. After surgery, the patient improved her language deficit, with persistent paraphasia, although with improvements in social communication and quality of life.

Nowadays, after 24 months of follow-up, the patient presents paraphasias and short-term memory disturbances with no additional neurological deficits, reaching the average survival rate reported in the literature for this entity. Appropriate seizure control was achieved after oncological surgery guided by ECoG (Engel IA). She is being treated with levetiracetam 1 g every 12 hours with a follow-up by the Department of Neurology, and she has had no seizures since surgery. The oncology department treated her with adjuvant radiotherapy (60 GY) in 30 fractions of the residual lesion with concomitant chemotherapy with temozolomide 120 mg daily.

## Discussion

Gangliogliomas is a well-differentiated, slow-growing glioneuronal neoplasm composed of a combination of neoplastic ganglion and glial cells that is characterized by genetic alterations that cause MAPK pathway activation. Previously, AGG was included in the WHO classification as a malignant subtype of GG. Currently, this term is excluded in the new 2021 WHO classification; however, it is mentioned that there are malignant variants of ganglioglioma that continue to be called anaplastic ganglioglioma in the absence of a new term. The WHO states that more studies are needed to confirm the existence of AGG through molecular studies and define clear diagnostic criteria. At the moment, since we do not have a clear term to refer to this type of high-grade version of GG, we choose to continue using the term AGG [3].

AGG in adults is an extremely rare entity. In a retrospective study of 326 primary intracranial gangliogliomas, they only found 17 cases that were classified as AGG [7]. They are rare tumors that can arise de novo or from a low-grade lesion with transformation, usually from the glial component [4].

The etiology and pathogenesis of this disease remain unclear; however, the cell of origin is noted to be a glioneuronal precursor [8,9]. The BRAF V600E mutation has been identified in 39% of cases and lacks prognostic significance. The incidence of this mutation appears to be inversely related to the age at presentation [4,10,11]. Multiple differential diagnoses and immunohistochemical analyses indicate that the morphological neuronal component is either positive for BRAF V600 E, H3K27M, or a loss of ATRX, representing a strong argument in favor of a mixed glioneuronal immunophenotype. CD34 immunoreactivity is also useful but is not specific to GG [4].

The WHO suggests that a molecular diagnosis looks for a BRAF p.V600E mutation or another change in the MAPK pathway, as well as a ganglioglioma methylation profile, ideally with a mutated IDH [3].

Histopathologic analysis can be challenging. GG are biphasic tumors composed of an admixture of neuronal and glial elements; they may be intermixed or geographically separate. Neuronal elements are composed of dysmorphic ganglion cells that may demonstrate abnormal clustering and lack of cytoarchitectural organization, cytomegaly, perimembranous aggregation of Nissl substance, or binucleated forms seen in <50% of cases. The glial component may resemble a fibrillary astrocytoma, an oligodendroglioma, or a pilocytic astrocytoma. They often demonstrate an infiltrative growth pattern microscopically and frequently include dystrophic calcification either within the matrix or as a neuronal or capillary incrustation. Focal cortical dysplasia may be associated with GG [3].

The immunophenotype neuronal markers such as MAP2, neurofilament, chromogranin A, and synaptophysin demonstrate the neuronal component. There is no specific marker to differentiate neoplastic neurons from their normal counterparts. GFAP and OLIG2 highlight the neoplastic glial cell component. Also, CD34 may be expressed in 80% of the GG, and it is not normally expressed outside of vascular endothelial cells in the mature brain. Ki-67 is typically <5% in GG, being higher in malignant variants (as in our patient), and it is limited in the glial component. VE1 antibody identifies BRAF p.600E mutant protein in gangliogliomas with this genetic alteration [3].

The imaging characteristics of gangliogliomas are varied; they can be diffuse, poorly delineated, or solid masses with remarkable heterogeneous enhancement. AGG, such as in this case, tends to be isointense or hypointense on T1-weighted and hyperintense on T2-weighted, and contrast enhancement is generally irregular [11]. MRI spectroscopy may reveal choline peaks with a decrease in N-acetyl aspartate [11,12].

GGs progress locally, and AGGs tend to progress distantly [13,14]. In our case, there is no evidence of progression to the neuroaxis. In a study of 19 pediatric patients with AGG, 10 patients experienced tumor progression, and of these, 5 progressed distantly [15]. These suggest that chemotherapy may be useful to reduce the risk of metastases to the neuroaxis.

In this case, the diagnosis was made based on the compatible histopathological and immunohistochemical analysis of a tumor with glial and neuronal components, as well as the patient´s clinical features and imaging study, which were also compatible. Likewise, because the lesion presented characteristics of malignancy both in the histological analysis (microvascular proliferation and mitosis) and in the immunohistochemistry (high Ki-67 index), we decided to diagnose it as an AGG, which is a term that, despite not being found in the current WHO classification, has been used to refer to the malignant variant of GG. It was not possible to perform the molecular analysis due to the lack of resources at the institution; however, we understand the need to perform such analysis to rule out differential diagnoses with other types of neoplasms.

Nevertheless, we report a rare case of an older adult woman. AGGs tend to occur at a young age, and most are males under 40 years of age. Patients with AGGs are associated with a poor survival rate (median about 24.7 months). The primary prognostic factor is resectability at presentation, with a median survival of 44 months in those with gross total resection (GTR) [2,6], which is difficult to achieve in eloquent areas. Awake brain surgery is preferred due to the possibility of brain mapping and performing a tumor resection as completely as possible without causing any neurological deficits. However, in our case, the surgery could not be performed with the patient awake because the patient failed the neuropsychological pre-surgical tests, and she was ruled out as a candidate for this modality of surgery. In this case, she underwent a subtotal resection to preserve memory and quality of life and was treated with concomitant adjuvant radiotherapy and chemotherapy. It was possible to obtain adequate control of the seizures as well as improvement in language, and a survival rate within the average described in the literature was achieved. The contemporary role of radiation and chemotherapy has not yet been established, and the efficacy of these treatments has yet to be determined [11]. In our opinion, adjuvant therapy seems to provide a greater survival rate, especially when there is a residual lesion.

## Conclusions

Anaplastic gangliogliomas are very rare tumors that can occur at any place in the neuroaxis. It is a poorly characterized entity with multiple differential diagnoses. Diagnosis based on morphological and immunohistochemical analysis, looking for the neuronal and glial components and characteristics of malignancy (mitosis, microvascular proliferation, necrosis, high Ki-67 index), is acceptable when no molecular analysis is responsible. The treatment of choice is GTR, or the greatest safe tumor resection possible, with adjuvant radiotherapy and chemotherapy. Follow-up must be carried out in conjunction with the neurology service for the management of epilepsy, as well as surveillance with periodic imaging studies of the neuroaxis. Further investigation is needed to unify the diagnosis and treatment of mixed malignant neuroglial tumors.
